# Recent Advances in Chemical Functionalization of 2D Black Phosphorous Nanosheets

**DOI:** 10.1002/advs.201902359

**Published:** 2019-12-05

**Authors:** Shameel Thurakkal, Xiaoyan Zhang

**Affiliations:** ^1^ Division of Chemistry and Biochemistry Department of Chemistry and Chemical Engineering Chalmers University of Technology Kemigården 4 SE‐412 96 Göteborg Sweden

**Keywords:** ambient stability, black phosphorous, chemical functionalization, nanosheets

## Abstract

Owing to their tunable direct bandgap, high charge carrier mobility, and unique in‐plane anisotropic structure, black phosphorus nanosheets (BPNSs) have emerged as one of the most important candidates among the 2D materials beyond graphene. However, the poor ambient stability of black phosphorus limits its practical application, due to the chemical degradation of phosphorus atoms to phosphorus oxides in the presence of oxygen and/or water. Chemical functionalization is demonstrated as an efficient approach to enhance the ambient stability of BPNSs. Herein, various covalent strategies including radical addition, nitrene addition, nucleophilic substitution, and metal coordination are summarized. In addition, efficient noncovalent functionalization methods such as van der Waals interactions, electrostatic interactions, and cation–π interactions are described in detail. Furthermore, the preparations, characterization, and diverse applications of functionalized BPNSs in various fields are recapped. The challenges faced and future directions for the chemical functionalization of BPNSs are also highlighted.

## Graphical abstract

Due to their fantastic properties, black phosphorous nanosheets (BPNSs) are an emerging 2D material bridging the gap between graphene and transition metal dichalcogenides. Chemical functionalization is an effective strategy in improving the ambient stability of BPNSs and to impart additional properties/functions. Herein, the latest developments of the chemical functionalization of BPNSs are summarized and the future directions are highlighted.

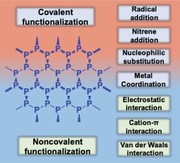

## 1 Introduction

**Figure 1:**
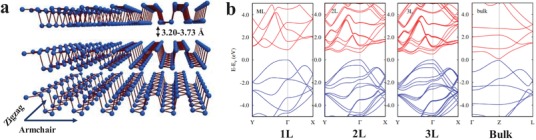
a) Schematic structure of three‐layer BPNSs. Reproduced with permission.^98^ Copyright 2015, John Wiley and Sons. b) Band structures obtained from DFT calculations for monolayer (1L), bilayer (2L), trilayer (3L), and bulk BP. Reproduced with permission.^99^ Copyright 2014, American Physical Society.

With the isolation of graphene in 2004, 2D materials have become a prime focus in material science research because of their extraordinary physicochemical properties such as large specific surface area, good mechanical strength, and high optical transparency.^1^, ^2^, ^3^, ^4^, ^5^, ^6^, ^7^, ^8^, ^9^, ^10^, ^11^, ^12^, ^13^, ^14^, ^15^, ^16^, ^17^, ^18^, ^19^ Inspired from the wide applications of graphene, a series of 2D layered materials such as transition metal dichalcogenides (TMDs),^20^, ^21^, ^22^, ^23^ hexagonal boron nitride (*h*‐BN),^24^, ^25^ graphitic carbon nitride (*g*‐C_3_N_4_),^26^, ^27^ layered metal oxides,^28^ layered double hydroxides^29^, ^30^ 2D polymers,^31^ transition metal carbides or carbonitrides (MXenes),^32^, ^33^ and elemental analogues of graphene^34^ have been established within the past few years. However, there are few inadequacies with these materials which limit their ideal performance in practical applications. For example, for field effect transistors (FETs), graphene is a zero‐bandgap semiconductor^35^ whereas TMDs have a tunable bandgap with a lower charge carrier mobility (10–100 cm^2^ V^−1^ s^−1^).^36^ Black phosphorous (BP), an allotrope of phosphorous, can be exfoliated into few‐layer nanosheets using the scotch tape based microcleavage.^37^ Monolayer BP shows a puckered structure along the armchair direction and a bilayer configuration along the zigzag direction (**Figure** 1a). The phosphorus atoms are chemically connected to each other through sp^3^‐hybridized covalent bonds within the layer and different layers of BP are stacked together by weak van der Waals interaction.^38^ BP shows thickness dependent direct bandgap (Figure 1b) from 0.3 eV (bulk BP) to 2.0 eV (monolayer), which is significantly larger than monolayer graphene and similar to TMDs (1.2–1.8 eV). Monolayer BP possesses a charge carrier mobility of 1000 cm^2^ V^−1^ s^−1^ at room temperature which is much larger than layered TMDs.^36^ As a result, BPNSs show an excellent on/off ratio of 10^3^–10^4^ in FETs and are considered as a promising material which can potentially bridge the gap between graphene and 2D TMDs.^39^, ^40^, ^41^ Importantly, compared to other 2D materials, BPNSs possess high in‐plane anisotropic properties due to their exceptional puckered structure.^42^, ^43^, ^44^, ^45^, ^46^, ^47^, ^48^, ^49^, ^50^ Because of these exciting properties, BPNSs are being utilized for various applications including photocatalysis,^51^, ^52^ biomedicines,^53^, ^54^ lithium and sodium ion batteries,^55^, ^56^, ^57^ lithium–sulfur batteries,^58^ supercapacitors,^59^, ^60^ FETs,^61^ optoelectronics devices,^62^, ^63^, ^64^, ^65^ sensing,^66^, ^67^, ^68^ etc.

Although BP is unique in many ways, chemical degradation of phosphorous into phosphorus oxides in the presence of ambient oxygen and water results in the rapid loss of semiconducting properties, due to the high reactivity of the lone pair electrons in BP.^69^, ^70^, ^71^, ^72^, ^73^, ^74^, ^75^, ^76^, ^77^, ^78^, ^79^ According to theoretical calculations, O_2_ molecule prefers a perpendicular configuration to approach the surface of BPNSs and tends to dissociate with exothermic energy of −4.07 eV per O_2_ molecule.^71^ The calculated lower dissociation barrier of 0.54 eV facilitates the oxidation readily at room temperature. After dissociation, atomic oxygen prefers a dangling configuration on the BP surface.^80^ Due to the high polar nature, H_2_O molecules prefer to bind the surface through hydrogen bonding to the lone pair of electrons in BPNSs. Based on calculations, H_2_O molecules will not dissociate on the surface directly as this is associated with a higher endothermic energy. However, for the oxidized BPNSs, the endothermic energy is significantly decreased, which enables dissociation of H_2_O on BPNSs surface easily.^71^ However, there are controversial reports on degradation, in which BPNSs can react with water even in the absence of oxygen.^67^ In addition to this, light irradiation can further speed up the degradation through photooxidation.^70^, ^81^, ^82^, ^83^ Based on ab initio electronic structure calculations and molecular dynamics simulations, the light induced ambient degradation of BPNSs involves three steps.^81^ In the initial stage, superoxide (O^2−^) is generated through a charge transfer reaction between BPNSs and oxygen under ambient light. In the next stage, the superoxide dissociates at the surface of BPNSs and forms two P—O bonds. Finally, water molecules interact with the oxygen atom in the P—O bond through hydrogen bonding, remove the O atom and its bonded P atom from the surface, and break the P—P bond connected to the bottom layer. In addition, an enhanced degradation was observed experimentally with the decrease in the thickness of BPNSs.^69^, ^84^ The bandgap of monolayer BPNSs matches with the redox potential of O_2_/O^2−^ and thereby, charge transfer rate from BPNSs to O_2_ is enhanced and thus leads to a higher oxidation rate.

To this end, continuous efforts have been devoted from researchers to overcome the poor ambient stability of BPNSs and various techniques such as Al_2_O_3_ protective layer coating,^85^, ^86^, ^87^, ^88^, ^89^ *h*‐BN encapsulation,^90^, ^91^, ^92^, ^93^ hybrid Al_2_O_3_/BN encapsulation,^94^, ^95^ ionophore coating,^96^ and chemical modification,^97^ have been demonstrated. For example, Al_2_O_3_ protection can preserve the properties of BPNSs over 7 d of ambient exposure, however, a long‐term stability is still challenging. Furthermore, in term of using BPNSs toward solution processing, chemical functionalization is more preferred and required. Chemical functionalization of BPNSs using various covalent and noncovalent approaches is an effective and controllable approach to passivate and modify the properties of BPNSs. For instance, chemical functionalization utilizes the lone pair of electrons present on the phosphorous atom to form direct chemical bonds, can thus, protect BPNSs away from oxygen, to achieve a long‐term stability for BPNSs at ambient conditions.

**Scheme 1:**
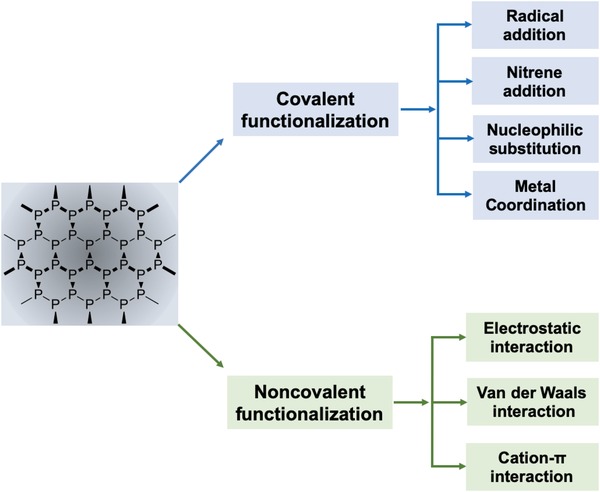
Chemical functionalization of BPNSs using covalent and noncovalent methods.

As the research on BPNSs continues to grow rapidly, critical reviews on the recent advances in various aspects of BPNSs are essential for further developments. Recently, Hui and co‐workers reported a review on the progress of preparation and electrochemical energy storage applications of 2D BPNSs.^100^ Furthermore, the advancements in different applications of BPNSs including photocatalysis, sensors, optoelectronics, photovoltaics and, biomedicines were also reviewed.^36^, ^39^, ^52^, ^57^ Considering the poor ambient stability of BPNSs, Abate and co‐workers reviewed different passivation techniques, particularly on inorganic coating and encapsulation methods.^77^ In addition, the review was also focused on anisotropic photophysical surface properties of BPNSs. Hirsch and co‐workers reviewed MoS_2_ and BP functionalization as post graphene chemistry with a general discussion on the reactivity of BP toward chemical functionalization.^101^ In this regard, there is an urgent need for a timely, comprehensive, in‐depth and critical review focusing on various latest development of chemical functionalization strategies of BPNSs, which hold a great potential not only for passivation but also for tuning the properties of BPNSs. Herein, an overview on the chemical functionalization of BPNSs based on various covalent and noncovalent approaches is presented (**Scheme** 1). For the covalent functionalization methods, different reaction mechanisms involving radical addition, nitrene addition, nucleophilic substitution, and metal coordination are described. In addition, various noncovalent strategies such as van der Waals, electrostatic, and cation–π interactions are also included. Preparation, characterization, and applications of functionalized BPNSs are also discussed. This timely review on the various chemical functionalization approaches will contribute to the future development of research in BPNSs.

## 2 Preparation of BPNSs

Mono‐ and few‐layer BPNSs can be prepared through both top‐down and bottom‐up approaches. In the top‐down method, bulk BP crystals are exfoliated into BPNSs by breaking the weak van der Waals interactions between stacked layers using external forces. Depending on the external force, the exfoliation can be mechanical exfoliation, liquid phase ultrasonic exfoliation, and electrochemical exfoliation. On the other hand, for the bottom‐up approaches, chemical vapor deposition (CVD) is the most common used method.

### 2.1 Top‐Down Methods

#### 2.1.1 Mechanical Exfoliation

BPNSs can be mechanically peeled off from bulk BP crystals using scotch‐tape.^37^ Although this method can produce high quality 2D BPNSs in a simple and cost‐effective manner, the obtained BPNSs show inhomogeneous size, poor repeatability, and extremely low production yield. To obtain BPNSs in a high yield, several alternative methods were employed including metal assisted mechanical exfoliation^102^ and exfoliation using polydimethylsiloxane (PDMS) stamp,^70^ viscoelastic stamp using blue Nitto tape,^103^ and poly(methyl methacrylate)/poly(vinyl alcohol) stack.^104^ In another approach, Zhu and co‐workers reported a large‐scale preparation of relatively stable BPNSs through high energy solid‐state mechanical ball milling of bulk BP in the presence of an additive LiOH.^105^ The high‐energy mechanical milling facilitates the generation of free radicals at the edges by breaking the P—P bonds and thus leads to the formation of stable hydroxyl functionalized BPNSs. In addition, this method has been widely utilizing as an efficient method to synthesize functionalized BPNSs for various applications.^106^, ^107^

#### 2.1.2 Liquid Phase Ultrasonic Exfoliation

**Figure 2:**
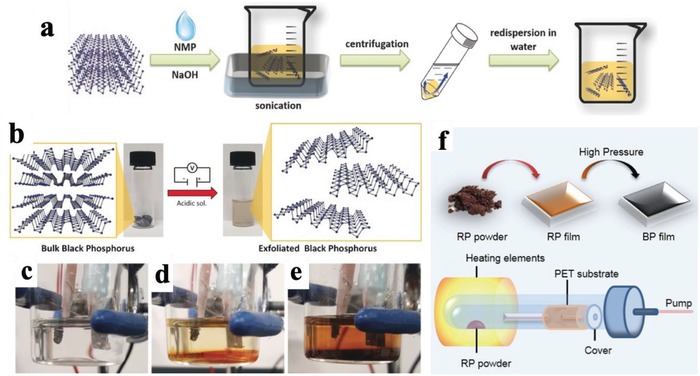
a) Schematic illustration of the fabrication process of NaOH‐NMP‐exfoliated BPNSs. Reproduced with permission.^98^ Copyright 2015, John Wiley and Sons. b) Electrochemical exfoliation procedure of BP in an acidic aqueous solution. The starting BP crystals (left) and the exfoliated BPNSs in DMF (right) are also shown. c) No potential applied. d) After 20 min of applying a voltage of +3 V. e) After 2 h of applied voltage. Reproduced with permission.^126^ Copyright 2017, John Wiley and Sons. f) Strategy for the synthesis of thin BP films using CVD and schematic apparatus for the deposition of red phosphorous (RP) film. Reproduced with permission.^135^ Copyright 2015, Institute of Physics (IOP) Publishing Ltd.

In this method, bulk BP is added into a solvent or solvent mixture, which is subjected to ultrasonication‐assisted exfoliation followed by centrifugation/standing by to yield mono‐ and few‐layer BPNSs. In this approach, the choice of solvent plays a key role in the yield and stability of the as‐exfoliated BPNSs. It was found that solvents with surface tension of 35–40 mJ m^−2^ facilitate the exfoliation of bulk BP.^108^, ^109^, ^110^ With respect to the solvent system, liquid phase exfoliation can be categorized into organic solvent exfoliation, water phase exfoliation, surfactant assisted exfoliation, polymer or ionic liquid (IL) assisted exfoliation. BPNSs dispersions can be prepared by exfoliation of bulk BP in organic solvents such as *N*‐methyl‐2‐pyrrolidone (NMP),^111^, ^112^ *N*‐cyclohexyl‐2‐pyrrolidone (CHP),^67^ γ‐butyrolactone,^113^ dimethylformamide (DMF),^114^ dimethyl sulfoxide,^114^ and isopropanol.^113^, ^115^, ^116^ Hersam and co‐workers have demonstrated that the size of BPNSs can be controlled by processing time and sonication power with NMP as the optimal solvent.^111^ In order to further improve the efficiency of exfoliation, several modified methods have been developed. For instance, the addition of NaOH in NMP results in a high yield exfoliation of BPNSs with a good stability in water (**Figure** 2a).^98^ Furthermore, the concentration of BPNSs in CHP by probe sonication instead of bath sonication can reach as high as 1 mg mL^−1^.^67^ Small organic molecules assisted liquid phase exfoliation can also produce high quality BPNSs for photocatalysts.^117^

In addition to organic solvents, water has been employed as a medium for exfoliation of BPNSs.^118^, ^119^ The surface tension of water can be tuned by adding surfactants into water. For example, Hersam and co‐workers utilized deoxygenated water with amphiphilic sodium dodecyl sulfate as the surfactant and achieved a higher concentration of BPNSs in water with flake thickness thinner than the ones produced using anhydrous organic solvent exfoliation.^120^ In another case, Peng et al. has reported polymer assisted exfoliation of bulk BP in polyvinylpyrrolidone solution.^121^ When bulk BP was exfoliated in ILs or polymer ionic liquids (PILs), mono‐ or multilayer BPNSs show high oxidation resistance at ambient conditions due to their interaction with ILs or PILs.^122^, ^123^ Although an improved stability is achieved, the high boiling point of ILs/PILs may hinder the application in surface deposition since it is not so easy to remove the ILs/PILs completely.

#### 2.1.3 Electrochemical Exfoliation

In electrochemical exfoliation, parameters such as electrolyte, operating voltage, and precursors, are very important for a successful exfoliation.^124^ Erande and co‐workers used bulk BP as working electrode and Na_2_SO_4_ as the electrolyte to yield BPNSs of 3–15 stacked layers.^125^ In the mechanism, oxygen and hydroxyl radicals (produced from the oxidation of water) were generated by the positive bias on the working electrode and inserted between adjacent layers of bulk BP, thus reducing the interlayer interactions. Subsequently, BPNSs were separated out from the bulk BP when the free radicals were oxidized to generate oxygen. The obtained yield of BPNSs is high (>80%), indicating that this approach can be potentially applied to large scale production. Modified electrochemical exfoliation approaches have been demonstrated by Pumera and co‐workers using bulk BP as working electrode, Pt foil as the counter electrode, and H_2_SO_4_ as the electrolyte (Figure 2b–e).^126^ Huang et al. have reported an electrochemical cation (tetrabutylammonium) insertion method to obtain few‐layer BPNSs.^127^ The number of layers can be controlled by adjusting the applied potential. Feng and co‐workers have reported an electrochemical delamination strategy to produce BPNSs: the one using *tetra*‐*n*‐butylammonium cations and bisulfate anions can have an exfoliation yield up to 78% and flake sizes on a micrometer scale.^128^ Despite progress has been made,^129^, ^130^, ^131^, ^132^, ^133^, ^134^ it is still challenging to prepare BPNSs mainly consisting of monolayer flakes through electrochemical exfoliation approach, which requires a better understanding of the mechanism of electrochemical exfoliation.

### 2.2 Bottom‐Up Method

CVD is a well‐established bottom up technique for fabricating large area layered materials.^136^, ^137^, ^138^, ^139^, ^140^, ^141^, ^142^ Ji and co‐workers have demonstrated an in situ CVD approach to fabricate BPNSs with a thickness of four layers and average areas of >3 µm^2^. In their method, amorphous red phosphorous (RP) thin film was first grown on a silicon substrate by heating RP powder or bulk BP at 600 °C. Then both high temperature and high pressure were applied to convert the RP thin film to BPNSs.^143^ Similar method was reported by Xia and co‐workers, RP film was thermally evaporated onto a flexible polyethylene terephthalate substrate and then converted into BP nanofilm under high pressure at room temperature (Figure 2f).^135^ Although CVD method has been successfully used to fabricate 2D nanomaterials including graphene, TMDs, and *h*‐BN,^140^, ^144^, ^145^ the progress for the synthesis of BPNSs using CVD approach is still in the early stage and more efforts are required to grow much thinner BPNSs. Another widely used bottom up method is the direct chemical synthesis of 2D materials from small precursors by hydrothermal, solvothermal, or templated synthesis methods.^146^, ^147^, ^148^, ^149^, ^150^ This method provides an alternative way to synthesize/prepare 2D materials on a large scale and in a relatively low‐cost manner. Recently, partially oxidized BPNSs have been prepared through a one‐step solvothermal approach by reacting white phosphorus in ethylenediamine at 100 °C for 12 h.^151^ However, this is an largely unexplored area in the preparation of BPNSs, more suitable precursors and synthetic methods are highly demanded for the efficient and cost‐effective synthesis of BPNSs.

Although various top‐down and bottom‐up approaches have been developed, large scale production of high quality BPNSs with precise control over the number of layers is still lacking. Therefore, development of more powerful synthetic methods for producing high quality BPNSs is required for further research progress in this field.

## 3 Characterization of BPNSs

**Figure 3:**
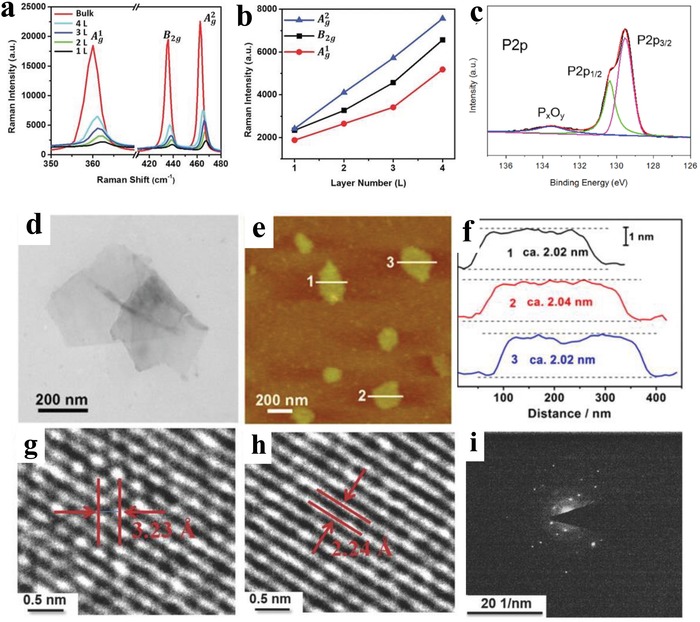
a) Raman spectra of bulk BP and BPNSs with different numbers of layers. b) Layer‐dependent Raman peak enhancement. Reproduced with permission.^98^ Copyright 2015, John Wiley and Sons. c) XPS P2p peaks of BPNSs. Reproduced under the terms of the Creative Commons Attribution 4.0 International License.^159^ Copyright 2017, The Authors, Published by Springer Nature. Morphology of few‐layer BPNSs: d) TEM image, e) AFM image, and f) corresponding height image. Reproduced with permission.^119^ Copyright 2015, American Chemical Society. g,h) HRTEM images with different crystal lattices and i) SAED patterns. Reproduced with permission.^98^ Copyright 2015, John Wiley and Sons.

As with other 2D materials, the quality of BPNSs can be characterized using various spectroscopic and microscopic techniques. Raman spectroscopy is a very useful and powerful technique for the characterization of BPNSs.^152^, ^153^, ^154^ The Raman spectrum of BPNSs exhibits three peaks located at 361, 437, and 465 cm^−1^, which are assigned to out‐of‐plane phonon mode (A^1^_g_), in‐plane modes along the zigzag direction (B_2g_), and in‐plane modes along the armchair direction (A^2^_g_), respectively (**Figure** 3a).^105^, ^155^ The Raman peaks of BPNSs are thickness dependent and show redshifts with increasing layer number, especially for the in‐plane mode A^2^_g_.^98^ In addition, the intensities of the three vibrational modes increase with layer number (Figure 3b). The intensity ratio of A^2^_g_ to A^1^_g_ was found to be higher for monolayer than multilayer BPNSs.^156^ The ratio of A^1^_g_/A^2^_g_ is highly indicative for oxidative status of BPNSs and shows an exponential decay with air exposure time,^69^ which is also recapped afterward in the chemical functionalization part.

X‐ray photoelectron spectroscopy (XPS) is often used to determine the chemical composition of BPNSs, for example, the presence of elements and functional units. In the high resolution P2p spectra, BPNSs show two intense peaks centered at 129.6 and 130.4 eV correspond to P2p_3/2_ and P2p_1/2_ signals of P—P bonds and a weak broad peak centered at 134.3 eV from PO*_x_* species due to the oxidation of BPNSs (Figure 3c). BPNSs typically show a 15–20% oxide shoulder with respect to the P2p peak corresponding to the P—P bonds.^67^ Fourier‐transform infrared spectroscopy (FTIR) measurements can provide information about functional groups present on BPNSs, which show two weak vibrational peaks at 1183 and 1005 cm^−1^ due to the presence of P=O and P—O groups from surface oxidation.^157^ When BPNSs are further functionalized, the foreign functional groups attached/bounded onto BPNSs can show distinct signals in the FTIR spectrum to help with the structure analysis. ^31^P NMR spectroscopy is another possible characterization tool for BPNSs. The characteristic peak for BPNSs is at ≈ 20.15 ppm ascribed to the P—P bond.^158^ The peak position can change when BPNSs are chemically modified,^107^ which will be discussed later in the chemical functionalization part.

The morphology and crystallinity of BPNSs can be examined by transmission electron microscope (TEM) and selected area electron diffraction (SAED) measurements (Figure 3d,g–i). The characteristic lattice parameters for BPNSs are about 3.23 Å and 2.24 Å, corresponding to the (012) and (014) plane of BP crystal, respectively.^160^ The SAED pattern indicates the highly crystalline nature of the BPNSs. Besides TEM, scanning electron microscope can be also used to study the morphology of BPNSs, possibly with the combination of elemental mapping using energy dispersive X‐ray (EDX).^161^ Thickness and size of BPNSs can be investigated by atomic force microscopy (AFM). The thickness of a monolayer phosphorene is considered to be 0.53 nm.^160^ However, in practice the thickness may vary depending on the substrate used and whether it has been oxidized (Figure 3e,f).^162^ AFM can provide further information about surface functionalization on BPNSs (an increase in thickness) and oxidative degradation by the loss of smooth surface morphology.^97^, ^162^, ^163^, ^164^

**Figure 4:**
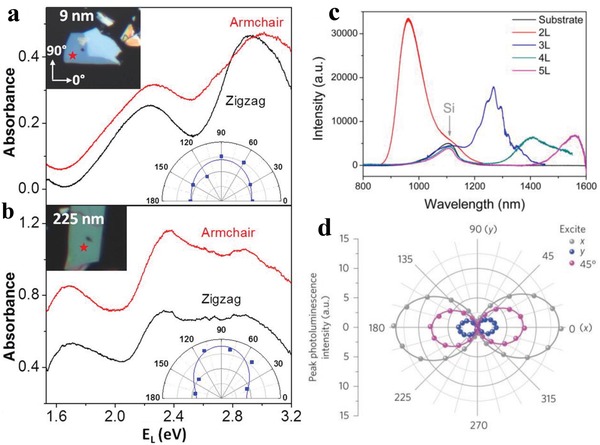
Typical absorbance spectra of a) a thin (9 nm) and b) a thick (225 nm) BP flake with incident light polarization along the armchair and zigzag directions. Reproduced with permission.^45^ Copyright 2015, American Chemical Society. c) Photoluminescence spectra of 2L, 3L, 4L, and 5L BPNSs. Reproduced with permission.^165^ Copyright 2014, American Chemical Society. d) Photoluminescence peak intensity as a function of polarization detection angle for excitation laser polarized along *x* (gray), 45° (magenta) and *y* (blue) directions. Reproduced with permission.^166^ Copyright 2015, Springer Nature.

Due to the thickness dependent bandgap energies, BPNSs have a wide range of UV–vis absorption spectra from visible to infrared region. BPNSs show highly anisotropic absorption properties originate from the symmetry forbidden selection rule.^166^, ^167^ Because of the anisotropic effect, BPNSs also exhibit dichroism which means the extent of absorption depends on the polarization state of incident light.^168^ It was found that BPNSs absorb polarized visible light more easily in the armchair direction with a larger absorption coefficient than in the zigzag direction (**Figure** 4a,b).^45^, ^169^ Because of the tunable bandgap, BPNSs also exhibit highly layer dependent photoluminescence from mid‐infrared to near‐infrared (NIR) wavelengths (Figure 4c).^165^, ^170^ Since the photoluminescence is originated from anisotropic excitons generated in BPNSs, the light emission is largely polarized along armchair direction (x direction in Figure 4d). In addition, the photoluminescence intensity also depends on excitation light polarization. Photoluminescence quenching effect was also observed for BPNSs when heterostacked with MoS_2_ or WS_2_.^171^

Besides the above techniques, thermogravimetric analysis (TGA) can be used to study the thermal stability of BPNSs and also determine the content of functional units in chemically modified BPNSs.^105^, ^172^ X‐ray diffraction (XRD) can reveal the crystalline structure and interlayer distance of (functionalized) BPNSs.^118^, ^173^

## 4 Covalent Functionalization on BPNSs

Various functional molecules/polymers or materials can be chemically attached onto BPNSs through direct P—C and/or P—O—C bond formation. So far, reactions involving different reactive intermediates, such as free radicals, nitrenes, and carbocations, have been reported for covalent functionalization of BPNSs. Formation of P—C and/or P—O—C bonds can not only passivate BPNSs but also potentially introduce new properties. On the other hand, covalent functionalization may largely alter the electronic properties of BPNSs due to direct breaking of P—P bonds.

### 4.1 Radical Addition

**Scheme 2:**
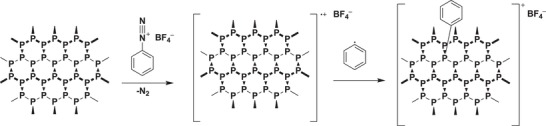
Mechanism of P—C bond formation on BPNSs using diazonium chemistry.

In the past, covalent functionalization of nanomaterials such as graphene, transition metal dichalcogenides, and carbon nanotubes using free radicals derived from diazonium salts has been well established.^174^, ^175^, ^176^, ^177^, ^178^ As in the case of BPNSs, radical intermediates can be generated using different methods including diazonium reaction, ball milling methods, and P—P bond breaking reactions. For example, diazonium salts can accept one electron from BPNSs and generate a highly reactive phenyl radical after release of nitrogen molecules. The radical subsequently added onto the surface of BPNSs to form a P—C covalent bond (**Scheme** 2).^97^, ^179^

#### 4.1.1 Reactions Using Diazonium/Diiodonium Salts

**Figure 5:**
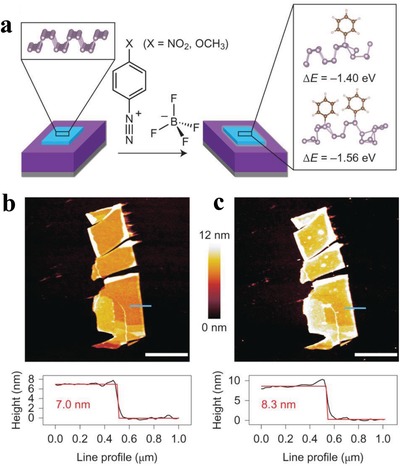
a) Reaction of benzenediazonium tetrafluoroborate derivatives and mechanically exfoliated few‐layer BPNSs (light blue) on a Si (gray)/SiO_2_ (purple) substrate. b) AFM image (top) of BPNSs prior to functionalization, along with the height profile extracted along the blue line (bottom). c) AFM image (top) of the same flake after 30 min of exposure to 10 × 10^−3^ m 4‐NBD. Reproduced with permission.^97^ Copyright 2016, Springer Nature.

Hersam and co‐workers have used diazonium chemistry for the covalent functionalization of BPNSs (**Figure** 5a).^97^ Few‐layer BPNSs were first prepared by mechanically exfoliating bulk BP on Si/SiO_2_ substrates and were then chemically modified with 4‐nitrobenzenediazonium (4‐NBD) and 4‐methoxybenzenediazonium (4‐MBD) tetrafluoroborate salts. From density functional theory (DFT) calculations, the formation of P—C bonds was found to be thermodynamically favorable with two aryl moieties per supercell of 16 phosphorous atoms. AFM measurements showed an increase of ≈1.5 nm in the height of the BP flake and an increased surface roughness upon functionalization (Figure 5b,c). 4‐NBD and 4‐MBD showed different conversion rates for the functionalization of BPNSs: the former is higher than the latter. In the P2p measurements, a broad peak at ≈133 eV corresponding to the P—C bonds was observed for 4‐NBD after 30 min of functionalization, whereas it was only evident after 180 min for 4‐MBD. Similarly, the doublet at ≈130 eV in the P2p spectra shows a more pronounced decrease in the intensity for 4‐NBD than for 4‐MBD after 180 min. From confocal Raman spectroscopy, the A^1^_g_ mode for BPNSs modified with 4‐NBD diminishes more rapidly than that for BPNSs modified with 4‐MBD. In the proposed reaction mechanism, a single electron transfers from BPNSs to the diazonium salts, leads to an elimination of N_2_ and the generation of reactive aryl radicals. The electron deficient radicals subsequently attack the basal BPNSs plane yielding the formation of P—C bonds. The electron transfer from the BPNSs to aryl diazonium ion is the rate‐limiting step for aryl diazonium reactions. The lower reduction potential of 4‐MBD relative to 4‐NBD results in a less‐favorable electron transfer, which is manifested in a slower P—C bond formation. After 3 weeks of ambient exposure, the aryl diazonium functionalized BPNSs exhibited an increased stability against oxidative degradation compared with the unmodified BPNSs. The chemical modification of BPNSs resulted in a controllable p‐type doping effect with an enhanced hole mobility and on/off ratio up to 10^6^ in FETs.

**Figure 6:**
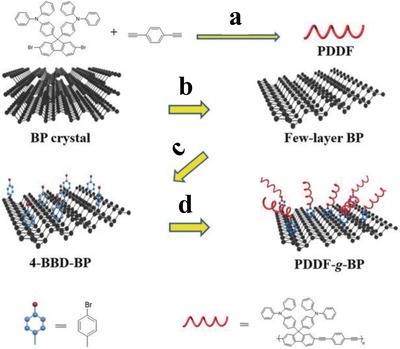
Synthesis of PDDF and PDDF‐*g*‐BP. a) Pd[(PPh_3_)_4_], CuI, Et_3_N, dry acetonitrile, 80 °C, 72 h. b) *N*‐methyl‐2‐pyrrolidone (NMP), ultrasonic radiation for 6 h (200 W). c) 4‐Bromobenzenediazonium tetrafluoroborate, tetrabutylammonium hexafluorophosphate, acetonitrile, room temperature (RT), 3 h. d) 1,4‐Diethynyl benzene, 9,9‐bis(4‐diphenylaminophenyl)‐2,7‐dibromofluorene, Pd[(PPh_3_)_4_], CuI, Et_3_N, dry acetonitrile, 80 °C, 72 h. Reproduced with permission.^180^ Copyright 2018, John Wiley and Sons.

In order to overcome the poor air stability and improve dispersibility of BPNSs, Chen and co‐workers synthesized a conjugated polymer derivative modified BPNSs, poly[(1,4‐diethynylbenzene)‐alt‐9,9‐bis(4‐diphenylaminophenyl)fluorene] (PDDF)‐covalently grafted BP (PDDF‐*g*‐BP), by using 4‐bromobenzene‐diazonium (4‐BBD) functionalized BP (4‐BBD–BP) (**Figure** 6).^180^ Initially, BPNSs with an average thickness of 10.4 nm were prepared by liquid phase exfoliation in NMP and then the 4‐BBD moieties were covalently attached to the BPNSs surface using diazonium chemistry. Then the PDDF‐*g*‐BP was synthesized by Sonogashira coupling reaction of 4‐BBD–BP and 1,4‐diethynyl benzene along with 9,9‐bis(4‐diphenylaminophenyl)‐2,7‐dibromofluorene. The formation of P—C bond was confirmed by the peak at 284 eV of C1s XPS spectra and IR frequency at 828.53 cm^−1^. The absence of Br3d signal at 71.5 eV in the wide scan XPS spectra of PDDF‐*g*‐BP suggests that PDDF was successfully end‐capped with 4‐BBD–BP to produce PDDF‐*g*‐BP. An electron transfer from PDDF to BPNSs was observed for PDDF‐*g*‐BP and was confirmed by steady state fluorescence studies. The photoinduced charge transfer was further explored using light induced electron paramagnetic resonance technique. A significant decrease in the intensity of the EPR signal was observed for the PDDF‐*g*‐BP after illumination, attributed to the formation of PDDF^∙+^—BP^∙–^ radical ion‐pair. Because of the improved solution processability and homogenous film formation, the Au/PDDF‐*g*‐BP/indium tin oxide (ITO) device showed a good nonvolatile memory performance with an on/off current ratio of 10^4^ compared to that of PDDF/BP blends. Interestingly, the on and off current was kept the same even after 200 cycles.

**Figure 7:**
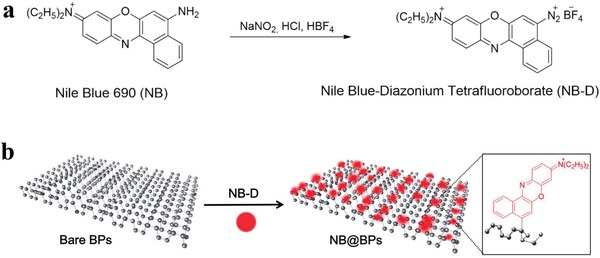
Schematic illustration of a) the synthesis of NB‐D and b) the preparation of NB@BPs. Reproduced with permission.^181^ Copyright 2017, American Chemical Society.

In another case, Yu and co‐workers described a modification strategy utilizing a fluorescent dye Nile Blue 690 via diazonium chemistry.^181^ The BPNSs with an average lateral size of ≈35.0 nm were prepared by liquid phase exfoliation in NMP. The diazonium tetrafluoroborate salt of the dye (NB‐D) was reacted with the exfoliated BPNSs to form stable P—C bonds on the BPNSs surface by aryl diazonium coupling (**Figure** 7). The NB@BPs showed a broad peak at 133.3 eV in the P2p XPS spectra and a peak at 284.2 eV in the C1s XPS spectra, corresponding to P—C bonds in the NB@BPs. The NB@BPs exhibited better photothermal performance and greater fluorescence intensity than the bare BPs, suggesting successful synthesis of fluorescent BPs by NB‐D covalent modification. This study paves the way to develop novel modified BPNSs with exciting properties by attaching functional molecules such as photo‐, electro‐, thermo‐, or piezo responsive materials and other functional dyes.^182^

**Figure 8:**
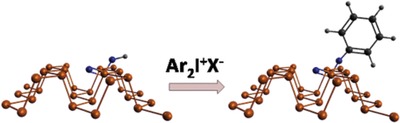
Covalent functionalization of BPNSs using aryl iodonium salts. Reproduced with permission.^183^ Copyright 2018, American Chemical Society.

Collines and co‐workers reported a covalent functionalization of liquid exfoliated few‐layer BPNSs using aryl iodonium salts and demonstrated superior ambient stability compared with arylation using diazonium salts.^183^ The highly electron deficient diaryliodonium salts have the ability to arylate both O and P nucleophiles by leaving aryl iodides, which enables covalent functionalization of BPNSs at room temperature with excellent ambient stability (**Figure** 8). The authors observed that the arylation using iodonium salts showed a greater degree of functionalization than the one using diazonium salts. The mechanism of the aryl iodide reaction with BPNSs is still not clear yet. In general, the P or O nucleophile can either react through a ligand exchange and then by a ligand coupling with the removal of aryl iodide leaving group, or through the radical mechanism as in the case of diazonium salts. In terms of stability, after 1 week of ambient exposure, the arylated BPNSs using aryl iodonium salt showed a 9% increase in the intensity of oxide peak while diazonium functionalized BPNSs showed a 30% increase in the intensity. Compared with diazonium salts approach, the enhanced stability of functionalized BPNSs using iodonium salts are attributed to the higher degree of functionalization of BPNSs. Iodonium salts can be applied to both aryl and alkyl groups providing an efficient route to covalent modification of BPNSs with increased ambient stability.

#### 4.1.2 Reactions Using Ball Milling Method

**Figure 9:**
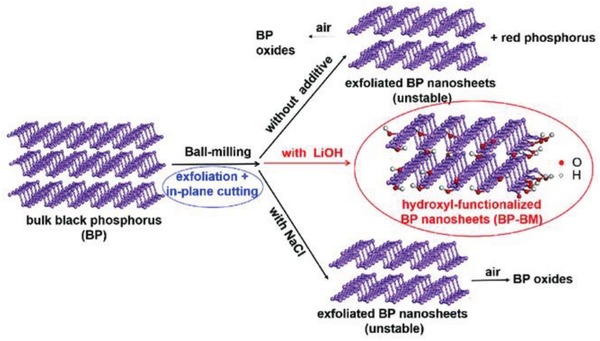
Scheme for the ball milling of BPNSs with additives (LiOH or NaCl) and without any additives. Reproduced with permission.^105^ Copyright 2017, John Wiley and Sons.

Solid state mechanochemical ball milling was established as a facile method for the exfoliation of graphite into few‐layer graphene nanosheets.^184^, ^185^, ^186^, ^187^, ^188^ Motivated from this, Yang and co‐workers have prepared stable few‐layer BPNSs (BP‐ball milling (BM)) by ball milling of bulk BP using anhydrous lithium hydroxide (LiOH) as an additive.^105^ In the absence of any noble metal cocatalyst, the BP‐BM exhibited significantly enhanced visible light photocatalytic H_2_ evolution rate (512 µmol h^−1^ g^−1^), much better than that of the bulk BP (18 times) and even higher than that of *g*‐C_3_N_4_. It was found that, the presence of LiOH additive is essential to obtain stable BPNSs and good H_2_ evolution activity. In contrast, ball milling without any additive or with an additive of NaCl, resulted in undesirable products such as BP oxides (**Figure** 9). The stabilization of BP‐BM compared to the bulk BP is due to the formation of edge selective hydroxyl functionalized BPNSs. The formation of hydroxyl groups involves the generation of reactive species such as free radicals via cleavage of P—P bonds during ball‐milling. The functionalization was confirmed by the high‐resolution O1s XPS spectra: the presence of an additional peak at 533.0 eV for BP‐BM is due to the P—OH bond, while the two intense peaks at 530.6 and 532.0 eV correspond to the P—O and P—O—P bonds, respectively. It should be pointed out that, the BP–BM photocatalyst was found to be relatively less stable due to the photooxidation of exfoliated BPNSs, which requires further functionalization strategy to increase the stability.

**Figure 10:**
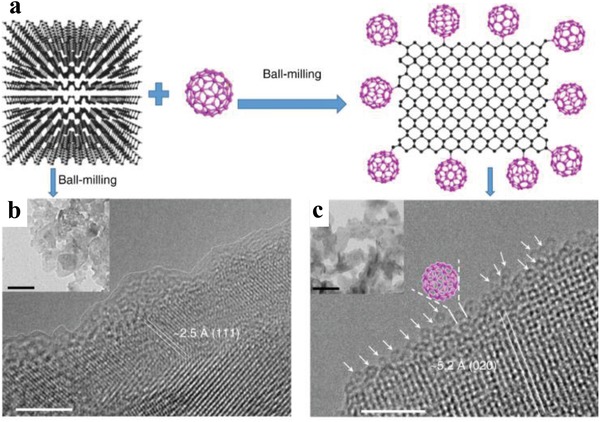
a) Schematic representation for the preparation of BP–C_60_ hybrid. HRTEM (scale bar: 5 nm) and TEM (inset, scale bar: 100 nm) images of the b) ball‐milled BPNSs and c) BP–C_60_ hybrid. Reproduced under the terms of the Creative Commons Attribution 4.0 International License.^107^ Copyright 2018, The Authors, Published by Springer Nature.

Chemical functionalization of BPNSs on the edges can largely preserve the intrinsic properties of BPNSs, while at the same time can potentially introduce new properties to BPNSs. As an example, edge selective functionalization of BPNSs by covalently bonding of C_60_ molecules was reported by Yang and co‐workers for the effective passivation of BPNSs without losing its surface integrity.^107^ The BPNSs–C_60_ hybrid was prepared using a facile one‐step solid‐state mechanochemical route by ball‐milling of bulk BP and C_60_ powders without any additives (**Figure** 10a). The average thickness of BP–C_60_ hybrid is ≈2.5 nm which corresponds to ≈4‐layer nanosheets. An average C_60_ molar content of 19 per 1000 P atoms was estimated from TGA. From XRD analysis, the crystal structure of BPNSs was found to be preserved after ball‐milling. In contrast to the ball milled BPNSs (Figure 10b), the TEM image of the BP–C_60_ hybrid shows lattice fringes of (020) plane at the edges of the BPNSs without any amorphous coating (Figure 10c), indicating that the BP–C_60_ hybrid possesses an improved structure stability at ambient condition. In addition, along the edges of the BPNSs, hollow nanospheres with diameter of ≈1.0 nm were observed in the high‐resolution TEM (HRTEM) image of the BP–C_60_ hybrid, which matches well with the morphology of C_60_ molecules. This indicates that C_60_ molecules are primarily attached at the edges of BPNSs in the BP–C_60_ hybrid. In scanning transmission electron microscopy energy dispersive X‐ray (STEM‐EDX) spectroscopic measurements of the BP–C_60_ hybrid, a much lower content of the C elements was found than the P elements. The presence of C_60_ moiety in the BP–C_60_ hybrid was further confirmed by FTIR spectra, showing four characteristic vibrational peaks of C_60_ at 526, 576, 1182, and 1428 cm^−1^. Besides, three additional vibrational peaks (707, 770, and 795 cm^−1^) attributed to the P—C bonds were only observed in the BP–C_60_ hybrid but not in the BP/C_60_ physical mixture. In the solid‐state ^13^C‐NMR spectra of the BP–C_60_ hybrid, an additional weak peak at 75.75 ppm was found due to the formation of sp^3^ carbon on C_60_, along with intense signals in the 130–160 ppm corresponding to the sp^2^ carbon of C_60_. The weak peak was absent in the spectra of both BP/C_60_ mixture and pure C_60_, suggesting the formation of sp^3^‐carbon on the C_60_ cage. The formation of the P—C bonds was further confirmed by the P2p XPS spectra with a new peak centered at ≈133.5 eV. The possible mechanism for the formation of the hybrid involves the generation of reactive species such as radicals at the edges via a mechanochemical cleavage of P—P bonds in the bulk BP during its exfoliation into few‐layer BPNSs using high‐energy ball milling. The activated C_60_ molecules were attached onto the activated edges of BPNSs via covalent P—C bonds. Notably, the degradation rate of BP–C_60_ in water was reduced by a factor of 4.6 compared with that of BPNSs. Since C_60_ molecules possess high stability toward light, oxygen, and water, the bonding of C_60_ molecules onto the edges of BPNSs can act as a sacrificial shield which effectively prevents BPNSs from the attack of light, oxygen, and water. The chemical attachment of C_60_ molecules in the BP–C_60_ hybrid results in a photoinduced electron transfer process from BPNSs to C_60_, which can suppress the recombination of charge carriers and subsequently enhance the photoelectric conversion property (ten times higher than that of the BP ball milling sample) and also the photocatalytic activity (much higher than the BP/C_60_ mixture).

#### 4.1.3 Reactions Involving P—P Bond Breaking

**Figure 11:**
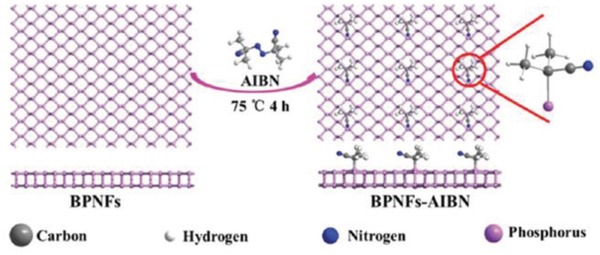
Scheme for the preparation of BPNFs–AIBN. Reproduced with permission.^158^ Copyright 2018, Royal Society of Chemistry.

In another strategy, BP nanoflakes (BPNFs) were covalently functionalized with carbon free radicals from azodiisobutyronitrile (AIBN) molecules.^158^ According to theoretical predictions, AIBN carbon free radicals are covalently attached to BPNFs through the breaking of P—P bonds (2.77 Å) and the formation of P—C bonds (2.01 Å) onto a supercell of 36 phosphorus atoms (**Figure** 11). The BPNFs‐AIBN with a size of 200–400 nm was prepared by reacting AIBN with BPNFs dispersed in a mixture of NMP and toluene (v/v 1:3) at 75 °C for 4 h under argon atmosphere. Compared with bulk BP, both the A^1^g and B_2g_ modes of BPNFs and BPNFs–AIBN do not display any changes, while the A^2^g mode showed a blueshift. In the FTIR spectra, the vibration frequencies of the cyano group in AIBN at ≈2242 cm^−1^ was redshifted to ≈2235 cm^−1^ after the functionalization reaction with BPNFs. The formation of P—C bonds was proved by the corresponding peak at −9.7 ppm in the ^31^P solid‐state NMR spectra. However, the peak intensity of the P—C bonds for BPNFs–AIBN was only 1.29%, suggesting that only few carbon free radicals were covalently attached onto BPNFs. In the XPS spectra, the appearance of a new peak at 133.1 eV attributed to the P—C bonds confirms the covalent functionalization of BPNFs. AFM and HRTEM measurements also indicated that the original morphology and crystalline state of BPNFs were retained after modification by carbon free radicals. Importantly, after functionalization, the stability of BPNFs in air and aqueous solution was significantly improved. However, besides the theoretical predictions, there is no direct experimental evidence that the P—P bonds were breaking during the reaction.

### 4.2 Nucleophilic Substitution

**Figure 12:**
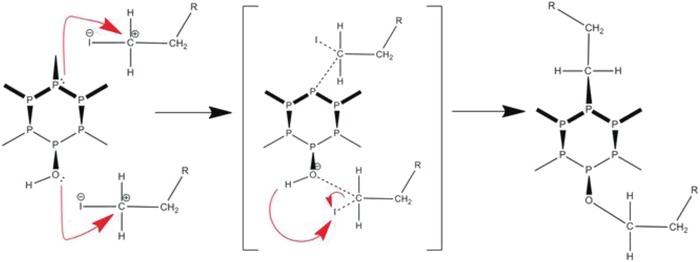
Proposed mechanism of the reaction on BPNSs through nucleophilic substitution with alkyl halides. Reproduced with permission.^157^ Copyright 2017, John Wiley and Sons.

Because of the lone pair electrons on the phosphorous atom, BPNSs can act as nucleophiles in the presence of electrophiles. Under certain conditions, oxygen atoms on hydroxyl groups introduced on BPNSs also make nucleophilic substitution possible. Toward this direction, Pumera and co‐workers have demonstrated covalent modifications of BPNSs to form P—C and/or P—O—C bonds based on nucleophilic substitution.^157^ In their approach, BPNSs were prepared by combination of ultrasonication and shear force milling in organic solvents under argon atmosphere. BPNSs can undergo direct reaction with alkyl halides (**Figure** 12). Alternatively, BPNSs can be first treated with thionyl chloride to form chlorinated BPNSs, which can be further subjected to reaction with aliphatic alcohols. The success of the nucleophilic substitution reactions was proved by XPS, Raman, and FTIR. For Example, the XPS spectra show evidence for the presence of long perfluorinated alkyl chains of nucleophilic reagents and formation of P—O—C bonds. In contrast, the application of highly reactive (S)‐bromomethyl ethanethioate led to the direct formation of the P—C bonds. Furthermore, the appearance of the new modes/bands in the Raman/FTIR spectra also suggests the formation of new bonds. Compared with nucleophilic substitution, other approaches like reaction with organometallic reagents and radical reaction with diazonium tetrafluoridoborates were found to give a lower degree of covalent functionalization.

**Figure 13:**
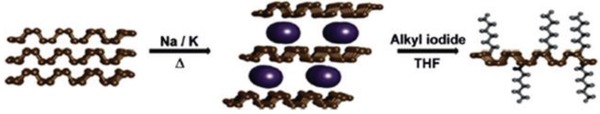
Reaction of BP intercalation compounds with alkyl halides through nucleophilic substitution. Reproduced with permission.^189^ Copyright 2019, John Wiley and Sons.

Taking advantages from the reductive graphene chemistry, Hirsch and co‐workers prepared BP intercalation compounds (BPICs) using alkali metals such as K and Na, which was then reacted with electrophilic alkyl halides to form P—C covalent bonds (**Figure** 13).^189^ The activated BPIC was dispersed in tetrahydrofuran (THF) by ultrasonication and then quenched with alkyl halides to yield the functionalized BPNSs as a dark gray powder. Statistical Raman spectroscopy showed a new band at about 145 cm^−1^ and additional new modes in the range of 250–300 cm^−1^, suggesting the formation of the P—C bonds. The same results were observed in the in situ experiments, in which hexyl iodide was slowly evaporated onto the synthesized BPICs under ultrahigh‐vacuum conditions. DFT calculations reproduce the above Raman bands and reveal the formation of radical species and P—P bond breaking in the BP lattice. Thermogravimetric analysis coupled to mass spectrometry (TG‐MS) measurements were performed: the two mass losses between 100 and 300 °C were assigned to the defunctionalization of the hexyl chain from the BP lattice and a sharp mass loss above 400 °C was due to the decomposition of BPNSs. The functionalized BPNSs showed a higher decomposition temperature compared to the pristine BPNSs due to the increased thermal stability. Importantly, the control experiments by functionalization of BPNSs with hexyl iodide without alkali metal intercalation under the same conditions did not show relevant features in the Raman and TG‐MS. Regarding to the stability, the functionalized BPNSs can be stable up to 15 d when exposed to oxygen and moisture. Quantitative magic‐angle spinning ^31^P solid‐state NMR measurements indicate that around 7% of negatively charged P atoms react quantitatively in the substitution reaction.

### 4.3 Nitrene Addition

**Figure 14:**
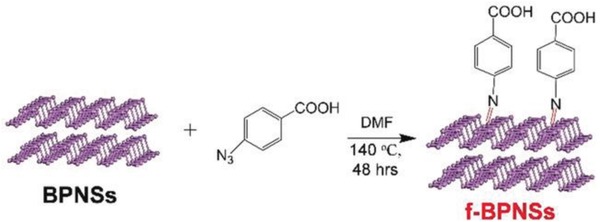
Reaction between liquid phase exfoliated BPNSs and 4‐azidobenzoic acid. Reproduced with permission.^155^ Copyright 2019, John Wiley and Sons.

In the diazonium chemistry, the formation of P—C single bonds retains one unpaired electron in the phosphorus atom, which may recede the passivation effect. To solve this problem, Yang and co‐workers reported the covalent azide functionalization of BPNSs, leading to the formation of P=N double bonds with phosphorus atoms and thus largely passivating the highly reactive BPNSs.^155^ In their method, BPNSs were prepared by liquid exfoliation of bulk BP in DMF and then mixed with 4‐azidobenzoic acid. The reaction mixture was then stirred in an inert condition at 140 °C for 48 h to yield the functionalized BPNSs (**Figure** 14). The reaction mechanism involves the in situ generation of nitrene as a reactive intermediate, which attacks the lone pair electrons on the phosphorus atom and results in the formation of the P=N bonds. The as‐prepared functionalized BPNSs were fully characterized by FTIR, solid‐state ^31^P NMR, XPS, with additional support from DFT calculations, indicating the existence of P=N double bonds. For example, the shoulder peak at −6.35 ppm in the ^31^P NMR spectra and the intense peak at 401.5 eV in the N1s XPS spectra are attributed to the P=N bonds. The morphology of both the BPNSs and functionalized BPNSs were studied by TEM/HRTEM and STEM‐EDX, suggesting that the crystallinity of BPNSs was remained after chemical functionalization. Importantly, through azide functionalization, the stability of BPNSs was significantly improved at ambient conditions.

### 4.4 Metal Coordination

**Figure 15:**
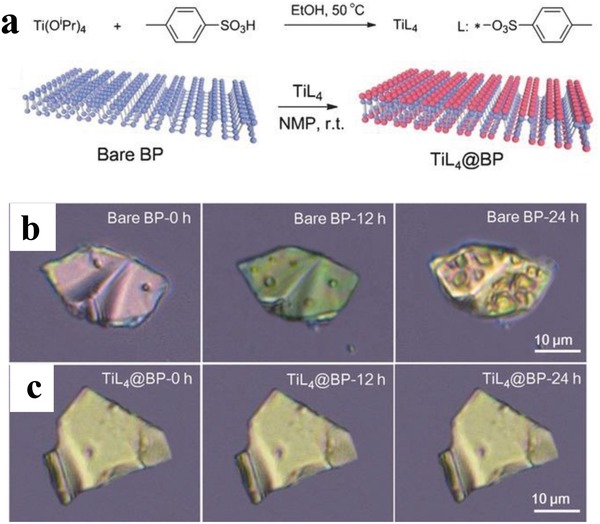
a) Synthesis of TiL_4_ and surface coordination of TiL_4_ onto BPNSs. Optical images of b) bare BP and c) TiL_4_@BP after exposure to the humid air at room temperature for different time period (12 and 24 h). Reproduced with permission.^190^ Copyright 2016, John Wiley and Sons.

The free electron pairs responsible for the oxidative degradation of BPNSs can be effectively protected by sharing with the vacant orbitals of electropositive metals or with electron deficient molecules, through coordination bonds. Chu and co‐workers have designed a titanium sulfonate ligand (TiL_4_, L referring to the sulfonic ester group) to react with BPNSs to form TiL_4_‐coordinated BP (TiL_4_@BP) to enhance the stability of BPNSs in water and humid air (**Figure** 15a).^190^ The TiL_4_ ligand was synthesized by reacting titanium tetraisopropoxide [Ti(O^i^Pr)_4_] with *p*‐toluenesulfonic acid in ethanol at 50 °C for 3 h. The ultrasmall BPNSs were prepared in NMP using a liquid exfoliation technique and the TiL_4_@BP with an average size of 3.3 nm was generated by reacting BPNSs with TiL_4_ in NMP at room temperature for 15 h. In the HRTEM image of TiL_4_@BP, the lattice fringes of 2.1 Å corresponding to the (014) plane of the BP crystal were observed. The ^1^H NMR spectra of TiL_4_@BP were found to be similar to those of TiL_4_ indicating successful coordination of TiL_4_ onto the BPNSs. The binding energy of P2p corresponding to the Ti—P coordination bond was found to be at 132.4 eV for TiL_4_@BP. For the TiL_4_@BP, the Ti2p_1/2_ (463.5 eV) and Ti2p_3/2_ (458.0 eV) peaks were detected, whereas no Ti2p peak was observed for the bare BP sample, confirming the effective functionalization of BPNSs. To evaluate the role of Ti coordination in the stability of BPNSs, the optical absorbance of TiL_4_@BP and bare BP were monitored. After 72 h, the absorbance of the bare BP at 450 nm was decreased by 55% compared to the original value, whereas the absorbance of TiL_4_@BP was maintained as high as 95%. In contrast to the severe degradation for the bare BP sample (Figure 15b), the optical images of TiL_4_@BP demonstrated that TiL_4_ coordination protects the BPNSs from oxidation in air with a relative humidity as high as 95% (Figure 15c). After TiL_4_ coordination, BPNSs maintain the photothermal performance even after 72 h. The TiL_4_@BP in a physiological environment exhibited excellent biocompatibility with different cells and good photothermal effect in killing the cancer cells when illuminated by an 808 nm laser. The present method provides a simple and efficient way to improve the stability of BPNSs against degradation at ambient conditions. Later on, the authors have employed TiL_4_@BPNSs and TiL_4_@BPQDs to regulate the aggregation of Amyloid‐β (Aβ), providing some insight into the development of functionalized BPNSs to prevent amyloidosis.^191^

**Figure 16:**
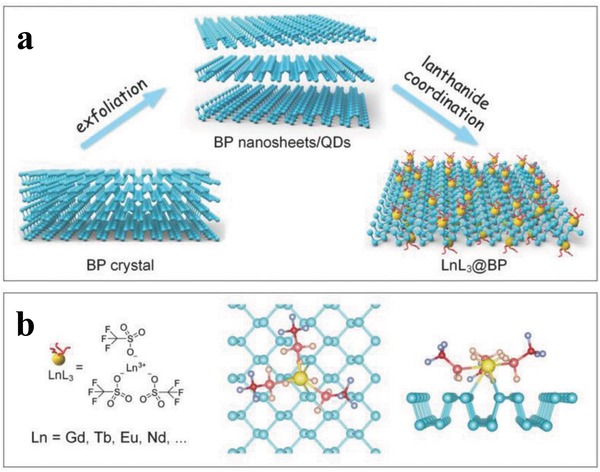
a) Schematic diagram for the synthesis of lanthanide functionalized BP nanosheets/QDs. b) The chemical formula of LnL_3_ and the atomistic model of the lanthanide ligand coordinated BP surface. Reproduced with permission.^192^ Copyright 2018, John Wiley and Sons.

Similarly, later on Yu and co‐workers used a lanthanide trifluoromethanesulfonates (LnL_3_) for the surface coordination of BP nanostructures.^192^ The electrophilic ligands strengthen the coordinating ability of Ln^3+^ and modification of BP nanostructures with LnL_3_ through coordination of the lone‐pair electrons of phosphorus with the empty d‐orbital of the lanthanide ions (**Figure** 16). The BP quantum dots (BPQDs) were prepared by ultrasonic liquid exfoliation method in NMP and then, the GdL_3_@BPQDs hybrid with a diameter of about 3.7 nm were prepared by stirring the BPQDs with excessive amount of GdL_3_ for 20 h under argon atmosphere. The HRTEM image reveals lattice fringes of 2.3 Å corresponding to the (014) plane of BP and energy‐dispersive X‐ray spectroscopy (EDS) analysis exhibited characteristic peaks of P, Gd, F, S, C and O. After GdL_3_ modification, the Raman scattering peaks was blueshifted and the zeta potential of BPQDs changes from −32.5 ± 1.4 to +28.6 ± 1.6 mV, confirming the efficient functionalization of BPQDs. Compared with GdL_3_, the binding energies of Gd4d_5/2_ and Gd4d_3/2_ of GdL_3_@BPQDs shift negatively by 1.5 and 1.1 eV, respectively and the binding energy of the P2p signal at 132.9 eV along with the normal BP peaks suggests the coordination of metal with phosphorus without influencing the BP structure. The effect of GdL_3_ coordination on the stability of BPQDs was examined by monitoring the absorption intensity where in contrast to BPQDs, the GdL_3_@BPQDs complex showed a minimal decrease in the absorption intensity after exposing to air for 8 d. The passivation is further confirmed by the P2p XPS spectra of GdL_3_@BPQDs, which is nearly unchanged under ambient conditions over 8 d. The surface coordination strategy was also successfully extended to BPNSs and BP microflakes and it is found that the present strategy can effectively passivate different types of BP from oxidation and degradation. The lanthanide functionalization of BP not only prevents the oxidation in water and humid air but also enables them to be fluorescent and possess high R_1_ relativities in magnetic resonance imaging (MRI). In a recent example, modified cisplatin—Pt—NO_3_ [Pt(NH_3_)_2_(NO_3_)_2_] was used for the surface modification of BPNSs to generate Pt@BPNSs, which was then interacted with DNA both in vitro and in vivo.^193^ Compared with the unmodified cisplatin, the Pt@BPNSs showed a good cellular uptake rate and dramatically improved the drug sensitivity of cisplatin‐resistant cancer cell lines, holding great potential for applications in photothermal/chemotherapy.

## 5 Noncovalent Functionalization of BPNSs

Covalent functionalization may largely change the intrinsic properties of BPNSs, such as the P—P bonds breaking. In order to preserve the pristine properties of BPNSs to a great extent, noncovalent functionalization of BPNSs has been developed by taking advantage of electrostatic interactions, van der Waals interactions, and cation–π interactions.

### 5.1 Electrostatic Interactions

Liquid phase exfoliated BPNSs can possess a negative zeta potential,^195^ which renders it possible to noncovalently functionalize BPNSs with positively charged species (e.g., small molecules, polymers) through electrostatic interactions.^195^, ^196^, ^201^, ^202^, ^203^, ^204^ For instance, Sabherwal and co‐workers employed nanostructured BP as a platform in an electrochemical sensor for the detection of Myoglobin (Mb), a cardiac disease biomarker. Stable few‐layer BPNSs were prepared by surfactant‐assisted liquid‐phase exfoliation of bulk BP in aqueous medium. The BPNSs were noncovalently functionalized with cationic polymer poly‐l‐lysine (PLL) to give PLL‐BPNSs. Negatively charged DNA aptamers for Mb were then immobilized onto the PLL‐BPNSs via Coulomb interactions between PLL and DNA. The sensor has a very low limit of detection of ≈ 0.524 pg mL^−1^ with a sensitivity of 36 µA pg^−1^ mL cm^−2^ for Mb spiked in serum samples. The sensor exhibited high specificity and sensitivity due to the high affinity screened aptamers and the enhanced electrochemical properties of the nanostructures, paving the way to achieve better cardiac biomarker detection for point‐of‐care diagnosis.

Jain et al. have utilized cetrimonium bromide (CTAB), an ionic surfactant with a long hydrophobic chain and nonbulky charged headgroups to prepare few‐layer BPNSs by liquid phase exfoliation in deoxygenated water.^195^ The ionic group present in the surfactants can interact with the lone pair electrons of phosphorous and the surfactant orient laterally at low concentration and vertically at high concentration over the BPNSs surface. This assembly resulted in a stable dispersion of BPNSs in water at highly concentrated surfactant solutions. The BPNSs exfoliated in the CTAB (P/CTAB) showed a smaller thickness of 3–10 nm in contrast to the BP nanostructures exfoliated in surfactant free deoxygenated water (P) and those in the presence of a structurally variant surfactant tetrabutylammonium hydroxide (P/TBAOH), indicating the favorable structure of CTAB for the efficient exfoliation of BPNSs in water. The higher redshifts (≈3.0−3.5 cm^−1^) of the A^2^g peak in the Raman spectra than that of P/TBAOH (0.5 cm^−1^) also confirmed the better intercalation and exfoliation capabilities of CTAB. In the XRD measurement, P/CTAB with vertically oriented CTAB molecules showed an increase in the interlayer distance between BPNSs compared with the BP nanostructures exfoliated in surfactant free deoxygenated water (P). Furthermore, DFT calculations suggested a strong and stable vertically oriented adsorption of CTAB on the BPNSs surface than the surfactant TBAOH possessing bulky alkane groups. Partial density of states of P/CTAB showed a decreased bandgap of 0.67 eV while for the pristine BPNSs the value is 0.82. Bader charge analysis revealed that noncovalent functionalization of BPNSs by CTAB led to a p‐type doping effect (BPNSs can donate 0.46 electrons to CTAB). The interaction between the surfactants and the BPNSs was investigated by NMR measurements. The retarded diffusion rate probed by 2D diffusion ordered spectroscopy, suggesting the presence of noncovalently bonded CTAB over the BPNSs surface. 2D nuclear overhauser effect spectroscopy (NOESY) further revealed the interdigitated arrangement of the CTAB surfactant. Importantly, the P/CTAB suppressed ambient degradation rate of BPNSs by 70−80% as indicated by XPS measurement.

**Figure 17:**
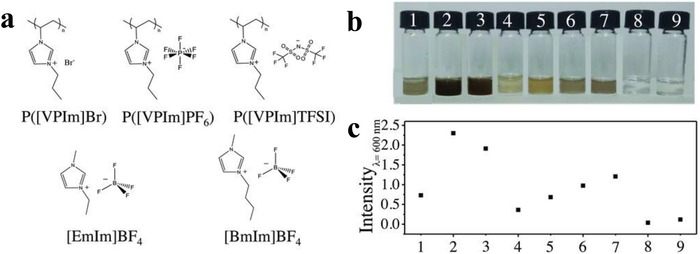
a) Chemical structures of the PILs and ILs. b) Photographs of BPNSs exfoliated with different solvents and c) comparison of their absorption intensity (wavelength at 600 nm). Reproduced with permission.^123^ Copyright 2018, John Wiley and Sons.

In another case, Zhang and co‐workers have synthesized highly stable functionalized BPNSs by exfoliating bulk BP in the presence of dilute solutions of PILs.^123^ The highly charged molecular chains of the PILs were wrapped onto the BPNSs through electrostatic interactions and acted as a shield to prevent BPNSs from air/moisture exposure. In contrast to exfoliation approach using pure ILs,^122^, ^205^ BPNSs can be produced in high yield and cost efficiency using diluted PILs solutions. Functionalization was carried out by ultrasonication of bulk BP in the PIL solutions namely P([VPIm]Br) in water, P([VPIm]PF_6_) or P([VPIm]TFSI) in DMF (**Figure** 17a TFSI refers to bis(trifluoromethane)sulfonimide). The extent of exfoliation was compared by exfoliating bulk BP in solvents alone (water or DMF), in pure IL monomers [EmIm]BF_4_ and [BmIm]BF_4_, and in aqueous solutions of [EmIm]BF_4_ and [BmIm]BF_4_. Dilution of pure monomer ILs with water resulted in significantly lower exfoliation yield compared with the one using pure monomer ILs, attributed to the reduced interactions between BPNSs and ILs. Compared with the above control samples, the exfoliation in the diluted solutions of PILs was significantly improved: using P([VPIm]TFSI can obtain the highest exfoliation concentration up to 0.19 mg mL^−1^ (Figure 17b,c). The ambient stability of the BPNSs was investigated by monitoring time‐dependent optical absorbance changes. After 7 d of ambient exposure, the decrease in absorbance for the unmodified BPNSs dispersed in water and DMF was 83.42% and 42.86%, respectively, whereas that of BPNSs/P([VPIm]Br) dispersed in water was 21.68%, and for BP/P([VPIm] PF_6_) and BP/P([VPIm]TFSI) dispersed in DMF the value was only 12.63% and 23.07%, respectively. In addition, after 21 d, the PILs modified BPNSs showed a A^1^_g_/A^2^_g_ ratio of 0.6 indicating a low oxidation degree of BP planes.^69^ XPS analysis further confirms the successful noncovalent functionalization and passivation of BPNSs. After 8 d of exposure in air, the intensity of P—O bond in the PILs functionalized BPNSs was found to be much weaker than that of the BPNSs, suggesting the good function of PILs in protecting BPNSs against ambient degradation. TEM images of the PILs modified BPNSs showed smooth surface and sharp edges over a period of 100 d suggesting highly crystalline structure with reduced oxidation. The retention of the high crystalline quality of the PILs modified BPNSs was further confirmed by SAED patterns. In contrast, a series of bubbles were observed on the surface of BPNSs/water and BPNSs/DMF samples after exposed to ambient conditions for a week, indicating the large extent of oxidation of BPNSs. Flexible photodetector devices based on the P([VPIm]TFSI) functionalized BPNSs showed good performances with a responsibility of 4.6 µA W^−1^ under a bias potential of 3 V and excellent device stability compared to the unmodified BPNSs based devices, demonstrating the great potential in the practical application of flexible optoelectronics. However, future efforts are required to further improve the on/off ratios (e.g., increasing the illumination intensity and fabricating photodetector devices with a narrower channel length).

### 5.2 van der Waals Interactions

Besides electrostatic interactions, recently there are also some reports on noncovalent modification of BPNSs by taking advantage of van der Waals interactions between BPNSs and foreign molecules.^206^ For example, redox active anthraquinone (AQ) has been used to noncovalently functionalize BPNSs, reported by Pumera and co‐workers.^199^ Few‐layer BPNSs with a thickness of 19.1 ± 9.7 nm were prepared by the shear exfoliation of bulk BP in aqueous surfactant sodium cholate, which was then modified by AQ to obtain the BPNSs–AQ complex. In the high‐resolution XPS spectra of the P2p, the signal corresponding to P oxides centered at about 134.1 eV is more intense for BPNSs than BPNSs–AQ suggesting that the BPNSs–AQ complex is more protected against oxidation. A relatively high abundance of C=O groups (≈19%) for the BPNSs–AQ in the XPS spectra of the C1s indicates the successful modification of BPNSs by AQ. After being exposed to ambient environment for 1 month, BPNSs exhibited much more extensive degradation than the BPNSs–AQ complex, indicating that noncovalent modification of BPNSs using the hydrophobic AQ can be a possible solution to reduce the degradation of BPNSs. Electrochemical measurements of the BPNSs–AQ complex showed that the AQ was stably immobilized onto the BPNSs and the redox peaks were stable over 100 cycles. The surface coverage of the AQ on the BPNSs was calculated to be ≈1.25 nmol AQ per mg of BPNSs and the electron transfer rate constant between AQ and BPNSs was 33 s^−1^. Interestingly, the BPNSs–AQ complex possessed a much higher gravimetric capacitance than the starting bulk BP material, holding potential applications in energy storage devices.

**Figure 18:**
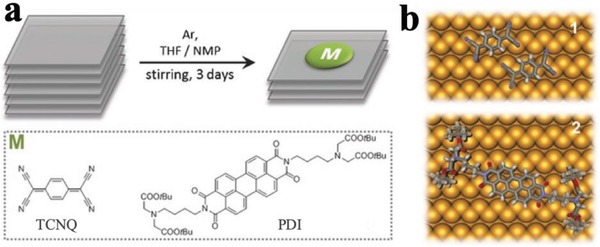
a) Functionalization of BPNSs using TCNQ and PDI. b) Schematic representation of TCNQ (top) and PDI molecules (bottom) adsorbed on BPNSs. Reproduced with permission.^200^ Copyright 2016, John Wiley and Sons.

In another case, as shown in **Figure** 18, BPNSs have been noncovalently functionalized with 7,7,8,8‐tetracyano‐*p*‐quinodimethane (TCNQ) or perylene diimide (PDI).^200^ The presence of TCNQ or PDI on the BPNSs was confirmed by several spectroscopic and microscopic characterization techniques, including attenuated total reflectance spectroscopy, Raman, AFM, scanning transmission electron microscope (STEM)–electron energy loss spectroscopy, UV–vis absorption spectroscopy, fluorescence emission spectroscopy, etc. For instance, upon excitation at 455 nm, the fluorescence emission of the PDI showed a dramatic quenching (≈66%) in the presence of BPNSs, suggesting there are interactions between PDI and BPNSs at the excited state. The mutual interactions between BPNSs and TCNQ/PDI was further studied using DFT calculations. Both the experimental and computational studies revealed that there were noncovalent interactions between the molecules and BPNSs, thus facilitating the exfoliation of bulk BP into few‐layer BPNSs. Electrons can transfer from the BPNSs to the TCNQ. The anchor of PDI on the BPNSs largely improves the stability of BPNSs at ambient conditions.

BPNSs can be effectively passivated to maintain their unique properties by formation of van der Waals heterostructures with other 2D materials such as graphene, TMDs, or *h*‐BN.^93^, ^207^, ^208^, ^209^, ^210^ For example, Doganov et al. prepared few‐layer BPNSs based heterostructures using graphene or *h*‐BN.^207^ BPNSs with a thickness of 3–9 nm were transferred onto SiO_2_/Si wafers using micromechanical exfoliation in an inert atmosphere. Then the heterostructures were fabricated by transferring graphene or *h*‐BN using a dry transfer method. The ambient stability was studied by AFM and Raman analysis on the covered and exposed areas of BPNSs based heterostructures. In contrast to the graphene or *h*‐BN protected surface, the exposed region of BPNSs showed a significant increase in the roughness over time. The optical images showed that, after 48 h of ambient exposure, the unprotected region of the BPNSs were completely degraded and no corresponding Raman bands of BPNSs were observed. On the other hand, the protected surface by graphene or *h*‐BN showed the three characteristics Raman peaks of BPNSs, along with Raman signals from the graphene or *h*‐BN. Compared with unprotected ones, the passivated BPNSs heterostructure based FETs showed 10 to 100‐fold improvement in electron mobility at room temperature. Remarkably, van der Waals interaction results in an in‐plane assembly of functional materials on BPNSs. Moreover, functionalization of BPNSs with other types of 2D materials including TMDs, layered metal hydroxides, metal organic frameworks (MOFs), 2D polymers, and functional organic molecules, is essential to further explore this hot area.

### 5.3 Cation–π Interactions

**Figure 19:**
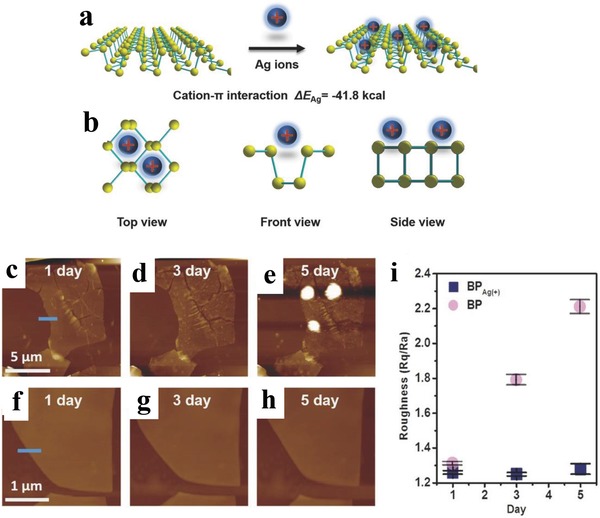
a) Schematic representation of adsorption of Ag^+^ on BPNSs. b) Three different views of the Ag^+^‐modified BPNSs. AFM images of c–e) the bare BPNSs and f–h) the Ag^+^‐modified BPNSs exposed to air for 1–5 d. i) Changes in the surface roughness before and after functionalization with exposure time. Reproduced with permission.^194^ Copyright 2017, John Wiley and Sons.

From the structure of BPNSs, the lone pair electrons on the phosphorus atoms are evenly distributed on the two sides of each BP layer and they can further interplay with each other to form conjugated π bonds, which can interact with metal ions through cation–π interaction.^194^ Yu and co‐workers employed metal ions such as Ag^+^ to interact with the exposed lone pair of electrons of BP forming Ag^+^‐modified BPNSs to enhance the stability against oxidation and degradation.^194^ The BPNSs were produced by a mechanical exfoliation method using scotch tape from bulk BP and were transferred to a Si wafer with a 300 nm thick SiO_2_ layer through a PDMS thin film. The samples were immersed in the NMP solution containing silver nitrate (1 × 10^−6^_M_) for 2 h, washed with NMP, and dried with argon gas to produce the Ag^+^‐modified BPNSs on the wafer. The metal ions can interact with the conjugated π bonds via cation–π interaction (**Figure** 19a,b). The combined energy between Ag^+^ ions and BPNSs was calculated to be −41.8 cal using DFT calculation, suggesting that free Ag^+^ can stably adsorb on the BPNSs surface to obtain the Ag^+^‐modified BPNSs. Then the Ag^+^‐modified BPNSs and bare BPNSs were exposed to air for 3 d to investigate their stability against oxidation. In the XPS spectra of P2p, BPNSs showed a strong peak at 134.0 eV corresponding to the PO*_x_* species, whereas this peak is not obvious for the Ag^+^‐modified BPNSs, indicating a better stability for the latter at ambient conditions. In addition, a new peak at 133.0 eV for the Ag^+^‐modified BPNSs was assigned to the interaction between Ag^+^ and BPNSs. In contrast to the bare BPNSs, the presence of a Ag3d_5/2_ peak at 367.8 eV for the Ag^+^‐modified BPNSs confirms the successful modification of Ag^+^ on the BPNSs surface. The ambient stability was further studied by AFM measurements (Figure 19c–i). The BPNSs and the Ag^+^‐modified BPNSs on the Si/SiO_2_ wafer were kept in air at a relative humidity of 95% and room temperature for 5 d. After 2 d, bubbles appeared in the bare BPNSs surface whereas the surface morphology of the Ag^+^‐modified BPNSs was preserved: after 5 d, no obvious bubbles, corrosions, or degradations were observed. The Ag^+^‐modified BPNSs FETs showed a hole mobility of 1666 cm^2^ V^−1^ s^−1^ and an on/off ratio of 2.6 × 10^6^, which is more than two times and 44 times higher than that of the bare BPNSs FETs, respectively. Based on DFT calculations, Ag^+^ modification can enhance the initial current of the hole‐transport, suppress the electron transport, lower the off‐state current, and increase the on/off ratio. The current modification strategy can be extended to other metal ions including Fe^3+^, Mg^2+^, and Hg^2+^, demonstrating an efficient approach to improve both the stability and transistor performance of BPNSs.

**Table** 1 summarizes and compares the preparation methods of BPNSs, various types of reactions/interactions used, reagents employed for functionalization and the stability of functionalized BPNSs. Obviously, they have shown different passivation effect in enhancing the stability of BPNSs upon exposure to ambient environment. The selection of chemical functionalization will reply very much on the applications: for example, if there is no requirement for the electrical performance of the BPNSs, covalent approaches involving P—P bond breaking can be considered.

**Table 1:** Summary of preparation, chemical functionalization, and stability of BPNSs

Refs.	Synthetic method of BPNSs	Functionalization	Type of reaction/interaction	Reagents for functionalization	Stability of functionalized BPNSs
97	Mechanical exfoliation on Si/SiO_2_ substrates	Covalent	Radical addition using aryldiazonium chemistry	4‐Nitrobenzenediazonium and 4‐methoxybenzenediazonium tetrafluoroborate salt	Stable up to 25 d of ambient exposure in solution
180	Liquid exfoliation in NMP	Covalent	1) Radical addition using aryldiazonium chemistry 2) Pd catalyzed polymerization	1) 4‐bromobenzenediazonium tetrafluoroborate 2) 1,4‐diethynyl benzene and 9,9‐bis(4‐diphenylaminophenyl)‐2,7‐dibromofluorene	Stable device performance even after 3 months ambient exposure
181	Liquid exfoliation in NMP	Covalent	Radical addition using aryldiazonium chemistry	Diazonium tetrafluoroborate salt of fluorescent dye Nile Blue 690	Stable after 3 d of ambient exposure in water solution
183	Liquid exfoliation in NMP	Covalent	Radical addition using aryldiodonium salt	Bis(4‐fluorophenyl)iodoniumtriflate, (perfluoro‐*n*‐propyl)‐phenyliodonium triflate and bis(4‐methylphenyl)iodonium hexfluorophosphate	Superior stability compared to aryldiazonium method after 1‐week ambient exposure
105	Mechanochemical exfoliation using ball milling	Covalent	Free radical/ions reaction via mechanochemical P—P bond cleavage in BP	LiOH	NA
107	Mechanochemical exfoliation using ball milling	Covalent	Free radical/ions reaction via mechanochemical P—P bond cleavage in BP	C_60_	The degradation rate of functionalized BPNSs was inhibited by a factor of 4.6
106	Mechanochemical exfoliation using ball milling	Covalent	Free radical/ions reaction via mechanochemical P—P bond cleavage in BP	Urea	Stable for 2 months in absolute ethanol; voltammogram in cyclic voltammetry remains unchanged for 1000 consecutive cycles
158	Liquid exfoliation in Isopropanol	Covalent	Free radical addition	AIBN	Oxidation degree was reduced by 17.82%
155	Liquid exfoliation in DMF	Covalent	Nitrene addition generated from organic azides	4‐Azidobenzoic acid	Stable after standing for 21 d in ambient conditions; stability is 4.7 times higher than that of diazonium‐functionalized BPNSs
157	Shear force milling in DMF	Covalent	Nucleophilic substitution reactions	Aliphatic alcohols or organometallic reagents	NA
189	Liquid exfoliation of BPICs in THF	Covalent	Nucleophilic substitution reactions	Alkyl iodides	NA
190	Liquid exfoliation in DMF	Covalent	Coordinating lone pair electrons of phosphorus atoms in the empty orbital of metal atom	Titanium sulfonate ligand	Stable after 1 week of ambient exposure in water
192	Liquid exfoliation in DMF	Covalent	Coordinating lone pair electrons of phosphorus atoms in the empty orbital of metal atom	Lanthanide sulfonate complexes	Stable after 8 d of ambient exposure in water
194	Mechanical exfoliation with scotch tape	Noncovalent	Cation–π interaction	Silver nitrate	Stable up to 5 d in ambient conditions and at least for 3 weeks in drying oven
195	Surfactant assisted liquid exfoliation in water	Noncovalent	Electrostatic interaction	Cetrimonium bromide (CTAB)	Degradation rate decreased significantly by 70−80%
196	Surfactant assisted liquid exfoliation in water	Noncovalent	Electrostatic interaction	PLL	Excellent storage stability of the developed aptasensor after 21 d
197	Liquid exfoliation in saturated NaOH solution in NMP	Noncovalent	Electrostatic interaction	Doxorubicin (DOX)	High photostability upon 808 nm laser irradiation for 5 min
123	PIL assisted liquid exfoliation in water or DMF	Noncovalent	Electrostatic interaction	P([VPIm]Br), P([VPIm]PF6), or P([VPIm]TFSI)	Stable up to 100 d
198	Liquid exfoliation in NMP	Noncovalent	Electrostatic interaction	Tripeptide Fmoc‐Lys‐Lys‐Phe	Stable for 2 d of ambient exposure
199	Shear exfoliation in aqueous surfactant sodium cholate	Noncovalent	van der Waals interactions	Anthraquinone	Stable up to 30 d
200	Chemical thinning in THF	Noncovalent	van der Waals interactions	Perylene diimide	Stable after 2 d of ambient exposure and stable up to 6 months in glove box storage
207	Mechanical exfoliation onto SiO_2_/Si wafers	Noncovalent	van der Waals interactions	Graphene or *h*‐BN	Stable up to 48 h at ambient conditions

## 6 Applications of Functionalized BPNSs

Due to the improved stability at ambient conditions, functionalized BPNSs have already shown potential applications in energy conversion and storage, electronic devices, biological field, nanocomposites, *etc*.

**Figure 20:**
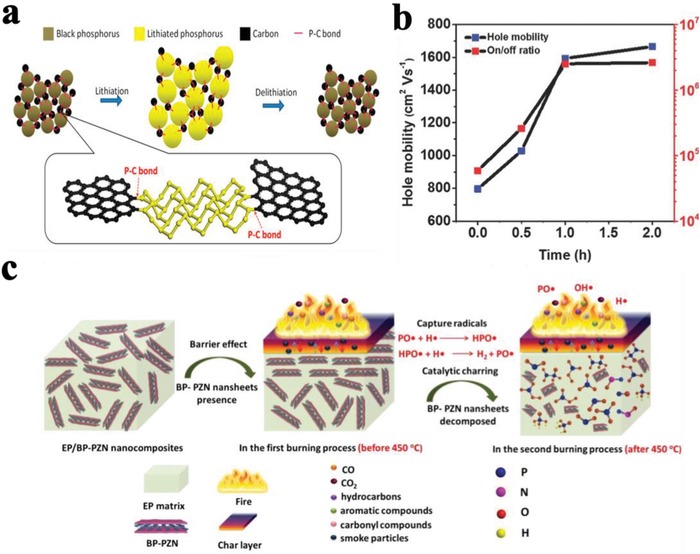
a) Schematic representation of lithiation and delithiation process in BP‐graphite composite. Reproduced with permission.^211^ Copyright 2014, American Chemical Society. b) Hole mobility and on/off ratio of BPNSs based FET device as a function of Ag^+^ modification time. Reproduced with permission.^194^ Copyright 2017, John Wiley and Sons. c) Schematic illustration of flame‐retardant mechanism for the epoxy resin (EP)/polyphosphazene‐functionalized BPNSs (BP‐PZN) nanocomposites during combustion. Reproduced with permission.^212^ Copyright 2019, American Chemical Society.

Based on their high specific surface area and good electrical conductivity, BPNSs have been already employed as electrode materials in energy storage devices.^60^, ^100^, ^115^, ^118^, ^131^, ^132^, ^213^, ^214^, ^215^, ^216^, ^217^, ^218^ The high theoretical capacity (2596 mAh g^−1^) of BPNSs makes it as a promising electrode material in lithium‐ion and sodium‐ion batteries (LIBs and SIBs).^61^ BPNSs based anode materials for LIBs exhibit improved first charge and discharge capacities compared to existing graphitic anode materials.^219^, ^220^ On the other hand, upon periodic lithiation and delithiation process, BPNSs show capacity decay due to the severe breakage of anode materials because of the large volume expansion. This problem can be tackled by chemical functionalization of BPNSs with carbon materials, the formation of P—C bonds makes BPNSs more stable during lithium insertion/extraction.^211^, ^221^ For instance, Cui and co‐workers synthesized a BPNSs–graphite composite using a high energy mechanical milling process (**Figure** 20a). The P—C bonds formed between BPNSs and graphite maintained excellent electrical contact and stability during lithium insertion/extraction process.^211^ The composite, as an anode material, exhibited an initial discharge capacity of 2786 mAh g^−1^ at 0.2 C and cycle life of 100 cycles with a capacity retention of 80%. Compared to lithium ion, due to its larger size, sodium ion can result in a higher volume expansion of BPNSs during sodiation process. To reduce this effect, BPNSs have been functionalized with different carbon materials including graphene, which can function as a cushion to maintain the structural stability of BPNSs and also as a conducting nanofiller to improve the electrical conductivity of BPNSs.^222^, ^223^ It was also demonstrated that covalently functionalized BPNSs with rGO could also improve cycle performance of BPNSs in SIB anode.^224^ In another report, a sulfur‐doped BPNSs‐TiO_2_ nanocomposite was prepared using a ball milling approach.^225^ The nanocomposite was then employed as anode material for SIBs and it showed good electrochemical performance: the discharge capacity can be up to 490 mA h g^−1^ over 100 cycles at 50 mA g^−1^, and it was maintained to be 290 mA h g^−1^ over 600 cycles at 500 mA g^−1^. Flexible all‐solid‐state supercapacitors using BPNSs‐carbon nanotube nanocomposite as electrode materials have been fabricated, showing a high power density of 821.62 W cm^−3^ and a stable electrochemical performance over 10 000 bending cycles.^226^

Since BPNSs showing thickness dependent bandgap, high carrier mobility and anisotropic transport, they have been employed as a channel material in FETs.^227^, ^228^, ^229^, ^230^, ^231^, ^232^ However, the poor ambient stability of BPNSs reduces their practical utilization in FETs. Effective passivation of BPNSs through chemical modification needs to be developed to achieve high performance functionalized BPNSs‐based FETs.^61^ As discussed in the previous sections, compared with unmodified BPNSs, both aryl diazonium functionalized BPNSs (covalent interaction) and Ag^+^ modified BPNSs (noncovalent interaction) show an improved ambient stability and an enhanced device performance in FETs (Figure 20b),^97^, ^194^ which is crucial for the realization of advanced electronic and optoelectronic devices.

For efficient usage of BPNSs with polymers for high‐performance nanocomposites, two important challenges have to be first addressed during processing: homogeneous dispersion of BPNSs in polymer matrix and strong interfacial interactions between BPNSs and polymer matrix.^233^, ^234^, ^235^, ^236^ One of the promising solutions to tackle the challenges is to chemically functionalize BPNSs, which can not only improve the dispersibility of BPNSs but can also reinforce the interfacial interactions, leading to a better chemical compatibility with different media and interfaces. It is worth pointing out that the functional units and polymers can potentially protect BPNSs away from degradation at ambient conditions. For example, recently Hu and co‐workers have prepared cobaltous phytate‐functionalized BPNSs and mixed them with polyurethane acrylate (PUA).^237^ The nanocomposite shows an increasement in the mechanical properties of PUA: an enhancement of tensile strength by 59.8% and tensile fracture strain by 88.1%. For the flame retardancy of PUA, there is an obvious decrease in the heat release rate (by 44.5%) and total heat release (by 34.5%), and generation of less pyrolysis products including highly toxic carbon monoxide. Importantly, Raman measurement and XRD analysis indicate that the nanocomposite was stable at ambient conditions for 4 months due to the isolation and protection effect on BPNSs. In another report, they have also fabricated a crosslinked polyphosphazene (PZN)‐functionalized BPNSs through a polycondensation reaction between 4,4'‐diaminodiphenyl ether and hexachlorocyclotriphosphazene on the surface of BPNSs.^212^ Then the polyphosphazene‐functionalized BPNSs were incorporated into epoxy resin to investigate the flame‐retardant property and smoke suppression performance (Figure 20c). The epoxy resin nanocomposite with a loading of 2 wt% polyphosphazene‐functionalized BPNSs showed an obvious improvement in the flame‐retardant property, showing a decrease of 59.4% and 63.6% for the peak heat release rate and the total heat release, respectively, with a reduction in the diffusion of the pyrolysis products. Importantly, as proved by XRD and Raman measurement, the nanocomposite showed a good stability at ambient conditions over 4 months, due to the protection from the polymer matrix.

**Figure 21:**
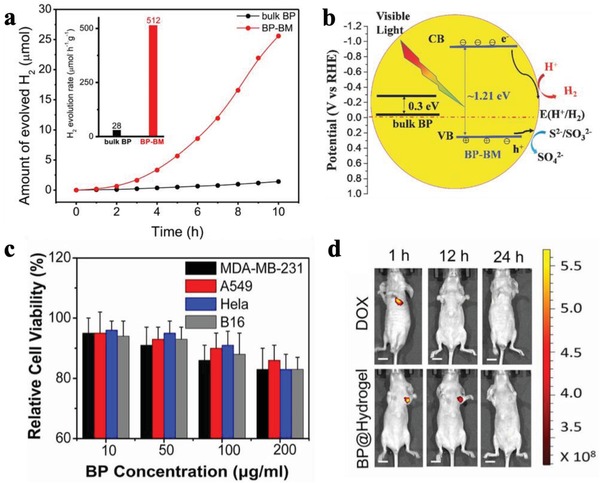
a) Photocatalytic activity of BP‐BM in H_2_ evolution reaction from water and b) the mechanism. Reproduced with permission.^105^ Copyright 2017, John Wiley and Sons. c) In vitro cell viability experiments of BP@Hydrogel. d) Fluorescence images of mice after the in vivo photothermal assay. Reproduced with permission.^250^ Copyright 2017, National Academy of Sciences.

As discussed in the introduction part, BP is a semiconductor with a layer‐dependent direct bandgap, facilitating a broad absorption in visible and NIR region, which renders BPNSs a good candidate as metal free photocatalyst.^238^ Computational calculations show H_2_ evolution process is possible as the conduction band (CB) of BPNSs is more negative than redox potential of H^+^/H_2_ (0 V vs normal hydrogen electrode).^239^ For example, as described in the above section, BP‐BM with negatively shifted CB and positively shifted valance band (VB) exhibited excellent H_2_ production efficiency (0.47%), which is higher than that of graphitic carbon nitride *g*‐C_3_N_4_ (0.1%) (**Figure** 21a). In contrast to bulk BP, the VB level of BP‐BM has been positively shifted which avoids undesirable recombination of the photogenerated electron–hole pairs (Figure 21b). In addition to this, so far, many other chemically modified BPNSs based photocatalysts have been successfully employed for H_2_ evolution reaction, revealing that BPNSs will be a good photocatalyst for energy production to solve the future energy crisis.^240^, ^241^, ^242^, ^243^, ^244^, ^245^, ^246^, ^247^, ^248^ Besides H_2_ evolution reaction, Co_3_O_4_ modified BPNSs have been used as an electrocatalyst for oxygen evolution reaction.^249^ The Co_3_O_4_ modified BPNSs showed a much better electrocatalytic performance than Co_3_O_4_ and BPNSs, attributing to the electron transfer process between the two species and improved stability of BPNSs. This study provides some useful insights to design highly efficient water oxidation electrocatalysts.

Due to their large surface‐to‐volume ratio, high thermal and electrical conductivity, and good optical properties, BPNSs can be potentially attractive toward biological applications.^251^ However, BPNSs suffer from degradation and gradually lose their properties when it is exposed to physiological environment, which is a great obstacle for their bioapplications. To passivate BPNSs and maintain its functions, effective functionalization strategies must be performed. The outstanding biocompatibility and biodegradability of functionalized BPNSs in physiological environment makes them attractive and promising for wide bioapplications in photodynamic therapy^197^ and photothermal therapy,^181^, ^201^, ^250^, ^252^, ^253^ tissue engineering,^254^, ^255^ biosensors, and bioimaging.^196^, ^202^, ^256^, ^257^ Qiu et al. have developed a new concept of light activation of BPNSs based hydrogel for cancer therapy.^250^ In their approach, BPNSs was prepared by liquid phase exfoliation in isopropanol. Then positively charged polyethylene glycol–amine was used to functionalize BPNSs through electrostatic interaction to form PEGylated BPNSs, in order to enhance their biocompatibility and physiological stability. Finally, BPNSs based hydrogel was prepared by mixing PEGylated BPNSs with a low‐melting‐point agarose. By tuning the light intensity and exposure duration, the release rates of doxorubicin (DOX) can be accurately controlled. In vitro and in vivo tests indicated that the BPNSs based hydrogel possessed an extremely good killing ability for cancer cell combined with tumor ablation effect (Figure 21c,d). The approach holds the potential to be transferred to practical research in curing cancer. In another report, Yang and co‐workers have reported tannic acid‐Mn^2+^ chelate networks on BPNSs,^258^ and the formed complex shows very good MRI contrast enhancement capability, excellent photoacoustic imaging performance, and high photothermal conversion efficiency, demonstrating great potential in imaging‐guided photothermal therapy. Importantly, balancing the stability and biodegradation of functionalized BPNSs is very important to achieve desirable biological functions with minimal residual during in vivo test.

Although functionalized BPNSs have shown interesting and promising results in the abovementioned areas, there is still a long way to go toward their large‐scale practical applications. For example, the toxic effect of functionalized BPNSs in biological systems should be always taken into considerations. For energy storage applications, improvement in electrical conductivity and cycle stability needs to be further investigated. On the other hand, functionalized BPNSs have been less employed in sensing, which can be one of the future directions to be explored.

## 7 Conclusions and Outlook

**Figure 22:**
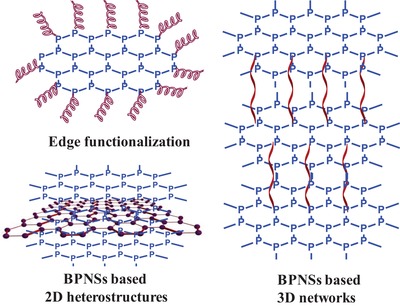
Schematic representation of different potential functionalized BPNSs including edge functionalized BPNSs, BPNSs based heterostructures and 3D networks.

The unique properties (tunable bandgap, high charge carrier mobility, and in‐plane anisotropy) of BPNSs make them an ideal bridge between graphene and TMDs. However, poor ambient stability of BPNSs hinders its possible wide applications. Chemical functionalization of BPNSs through covalent or noncovalent approach using metal oxide, functional organic molecules, other 2D materials or polymers have been proved to be a useful strategy to protect BPNSs from degradation at ambient conditions: it can not only passivate BPNSs but also introduce new properties to BPNSs. The reaction can be carried out in solid‐state or through liquid phase process. Depending on different methods used, they have shown different effectiveness on the passivation of BPNSs. For covalent functionalization, free radical reaction using diazonium compounds or aryl iodonium salts or ball milling, nucleophilic substitution to form P—C and/or P—O—C covalent bonds, has been successfully developed. In contrast to the above covalent approaches, nitrene addition results in a better stabilization of BPNSs owing to the utilization of both unpaired electrons present in the phosphorous atom. Coordination of the lone‐pair electrons on phosphorus atoms with metal complex is another effective way to stabilize BPNSs. On the other hand, noncovalent approaches such as electrostatic interactions, cation–π interactions, have been employed to prepare BPNSs based complex. Through van der Waals interactions, BPNSs can be combined with other type of 2D materials or functional molecules to fabricate novel heterostructures possessing extraordinary properties and performance.^208^, ^209^, ^259^, ^260^, ^261^, ^262^, ^263^, ^264^, ^265^, ^266^ Building BPNSs‐based heterostructures through solution processing is a promising route to prepare them in a large scale (**Figure** 22), however, achieving a highly uniform and ordered nanostructures is still challenging.^267^, ^268^, ^269^, ^270^, ^271^, ^272^ For example, a 2D BPNSs/platinum heterostructure has been developed and used as a highly efficient photocatalyst for solar‐driven chemical reactions.^268^ Compared with unmodified BPNSs, functionalized BPNSs exhibit improved performance in different applications such as LIBs/SIBs, photocatalysis, (opto)electronic devices, photothermal/photodynamic therapy, and sensing. However, there is still a long way to go before functionalized BPNSs can find their practical applications in various fields at ambient conditions.

Although rapid progress has been made on the chemical functionalization of BPNSs, the research area is still in its sprouting stage. There are several challenges to be resolved. The first prerequisite is to produce high‐quality BPNSs (with bigger sizes) in a large scale, which is yet to be resolved, largely relying on the further development of preparation methods. For example, other bottom‐up approaches such as hydrothermal/solvothermal method from molecule precursors needs to be developed.^151^ One of the biggest challenges in BPNSs research is still to find effective ways to minimize the degradation of BPNSs at ambient conditions both in the solid form and in solvent media. It should also be mentioned that the mechanism of degradation of BPNSs is still in debate and requires further efforts from both computational and experimental research to deeply understand the exact degradation mechanism and thus design novel strategies for effective passivation. Development of stable BPNSs is particularly demanded for their practical applications in various fields. Nevertheless, achieving a long‐term stability of functionalized BPNSs over months or years is currently still challenging. Efficient passivation could be achieved by developing new chemical functionalization strategies, for example, reacting/interacting effectively with the paired electrons on BPNSs using different kinds of functional units.^273^ This will largely broaden/enrich the chemistry of BPNSs. Special attention should be paid to that, for certain applications such as (opto)electronics devices, the intrinsic properties of BPNSs should be reserved at the most extent after chemical functionalization. For covalent functionalization, improving the selectivity (e.g., edges or defects or certain sites/domains selectively functionalized, Figure 22),^107^ will hold the potential to precisely control the properties of BPNSs. On the other hand, building functionalized BPNSs based 3D hierarchical nanostructures can be a useful way to prevent the aggregation of BPNSs and thus the properties of BPNSs can be fully utilized (Figure 22).^274^ Further issues regarding to how the charge transfer happens and how the charges are spatially distributed between BPNSs and functional units, still need to be fully understood at the fundamental level.^275^

Another major challenge is how to accurately determine the exact chemical structure of functionalized BPNSs through covalent modification, which is currently lacking direct evidence. This will rely very much on the development of advanced microscopic characterization techniques such as HRTEM, AFM, or scanning tunneling microscope, in order to be able to monitor the reactions in real time (in situ) and to “see” the true chemical structures of the functional units attached onto BPNSs. Additional techniques such as solid NMR or 2D NMR or MS can be also employed to determine the chemical structures or to probe the interactions between functional units and BPNSs.^195^, ^276^ Further support may come from theoretical calculations such as DFT, which will help to predict the chemistry, process, and mechanism.^157^, ^189^ It is reasonable to expect that, with the continuous effort in the research of chemical functionalization of BPNSs, this area will continue to grow in the coming years and more breakthroughs will emerge to realize their practical applications in various fields.

## Conflict of Interest

The authors declare no conflict of interest.
